# Implementing a Quality Improvement Collaborative to Improve Hypertension Control and Advance Million Hearts Among Low-Income Californians, 2014–2015

**DOI:** 10.5888/pcd14.160587

**Published:** 2017-07-27

**Authors:** Desiree R. Backman, Neal D. Kohatsu, Zhiwei Yu, Rachel E. Abbott, Kenneth W. Kizer

**Affiliations:** 1 California Department of Health Care Services, Sacramento, California; 2 University of California, Davis, Institute for Population Health Improvement, Davis, California

## Abstract

From January through December 2015, the California Department of Health Care Services, which administers Medi-Cal, the nation’s largest Medicaid program, conducted a quality improvement collaborative (QIC) with 9 Medi-Cal managed care plans (MCPs) aimed at improving hypertension control consistent with the Million Hearts initiative. The QIC included quarterly webinars and links to local, state, and national resources that consisted of materials and consultations with subject matter experts. Participating MCPs demonstrated an average increase of 5.0 percentage points in their rates of controlled hypertension. Collaboratives can achieve substantial quality improvement in Medicaid managed care plans.

## Objective

Hypertension is a major risk factor for deaths related to coronary heart disease (CHD) and stroke. Approximately one-third of adults in the United States have hypertension, but only about half (52%) have their hypertension under control (1). Million Hearts is a national initiative to prevent 1 million heart attacks and strokes by 2017 through strategies such as achieving blood pressure control among 70% of those who have hypertension (2).

The objective of this study was to determine the effect of a quality improvement collaborative (QIC) on hypertension control rates among 9 managed care plans (MCPs) whose performance at baseline was below the Million Hearts target.

## Methods

Approximately 14 million Californians are enrolled in Medi-Cal (3), which is administered by the California Department of Health Care Services (DHCS). Enrollees eligible for full-scope benefits (nearly 11 million) receive health care provided by a network of 23 MCPs (4). Ten MCPs, 9 of which performed below the Million Hearts target of 70% controlled high blood pressure, volunteered to participate in the QIC. The plan performing above the target was excluded from analysis.

In 2015, 407,911 Medi-Cal members enrolled in a MCP had been diagnosed with hypertension. More than 60% (246,206) of those with hypertension were enrolled in 1 of 9 MCPs that participated in the QIC (5). Of the 23 MCPs, the analysis was limited to QIC-participating and nonparticipating plans with continuous Controlling High Blood Pressure (CBP) data since 2009 (n = 19).

From January through December 2015, DHCS held quarterly, 1.5-hour webinars with MCPs in which local, state, and national leaders presented hypertension control best practices and provided evidence-based tools and resources (6,7). For example, the MCPs were exposed to an effective quality improvement program, the Right Care Initiative (8). Subject matter experts within DHCS also were available for consultation. The MCPs shared successes and challenges in controlling hypertension. MCPs also had access to a webpage providing useful resources. After each webinar, DHCS sent MCPs a survey to garner immediate feedback that was used to design and tailor subsequent webinars.

DHCS measures the effectiveness of its MCPs, in part, through the Healthcare Effectiveness Data and Information Set (HEDIS) (9). An external quality review organization collects and uses administrative and medical record data to calculate HEDIS rates for each MCP annually (10). The HEDIS CBP measure is defined as the percentage of members aged 18 to 85 years who had a diagnosis of hypertension and whose blood pressure was adequately controlled during the measurement year. The measure’s threshold for hypertension control in adults varies by age and diabetes status, ranging from less than 140/90 mm Hg to less than 150/90 mm Hg (11).

SAS version 9.4 (SAS Institute, Inc) was used to conduct an analysis of CBP rates, by MCP. The CBP rates were weighted on the basis of each plan’s number of enrollees. We used the *χ* ^2^ test to examine changes in performance (ie, percentage point difference) between calendar year 2014 and 2015. Significance was set at *P* < .05.

## Results

Since 2009, all QIC-participating MCPs had a downward trend in CBP rates. For that reason, managed care staff at DHCS strongly encouraged low-performing MCPs to participate in the QIC. In 2014, the CBP weighted average for all plans was 61.2%, and the average CBP rate among the 9 participating MCPs was 56.3% (range, 43.1%–69.3%).

During the QIC intervention year (2015), CBP rates improved significantly in 7 of 9 (78%) participating MCPs. The largest improvement was 14.6 percentage points, representing a 33.9% improvement in hypertension control (Table). The mean CBP rate improvement was 5.0 percentage points.

**Table:** Controlled High Blood Pressure Rates for Medi-Cal Managed Care Plans, Comparison of Calendar Year 2014 and 2015, HEDIS

Medi-Cal Managed Care Plan Name	Calendar Year 2014		Calendar Year 2015		Percentage Point Difference^b^	Percentage Change %
	Eligible Population	CBP Rate,^a^ %	Eligible Population	CBP Rate,^a^ %		
Alameda Alliance for Health	17,072	43.1	13,963	57.7	14.6	33.9
Anthem Blue Cross Partnership Plan	13,956	48.3	29,941	54.6	6.3	13.0
CalOptima	22,881	69.3	29,802	72.5	3.2	4.6
Care1st Partner Plan	2,913	59.4	3,796	54.0	−5.4	−9.1
Health Net Community Solutions, Inc	34,958	61.9	40,917	59.4	−2.5	−4.0
Health Plan of San Mateo	7,333	61.8	9,054	68.9	7.1	11.5
LA Care Health Plan	53,672	66.8	82,695	68.3	1.5	2.2
Molina Healthcare of California	12,147	44.5	17,829	53.3	8.8	19.8
Partnership Health Plan of California	16,461	52.1	18,209	63.3	11.2	21.5

Abbreviations: CBP, controlled blood pressure; HEDIS, Healthcare Effectiveness Data and Information Set.

a The CBP rates were weighted on the basis of the size of each plan’s eligible population.

b All significant at *P* < .05; *P* values determined by using χ^2^ test.

Ten MCPs did not participate in the QIC. CBP rates declined in 6 of the 10 nonparticipating MCPs from 2009 to 2014. Four were trending upward since 2009, although 1 of the 4 plateaued in 2011. In 2014, the average CBP rate among nonparticipating MCPs was 64.8%. In 2015, their average rate decreased by 5.7 percentage points to 59.1%. In addition, during the study period, CBP rates decreased in 9 of the 10 nonparticipating MCPs.

## Discussion

Participation in the QIC was associated with improved hypertension control; 7 of 9 MCPs demonstrated improved control in the intervention period compared with baseline. This improvement was notable because all plans had a multiyear history of declining hypertension control rates before the intervention. In addition, among nonparticipating plans, 9 of 10 showed decreasing hypertension control rates during the study period. These findings show that there was not a general, secular trend of hypertension control improvement among MCPs.

Several factors may explain the positive association. First, there were high participation rates for each of the quarterly 1.5-hour webinars. Second, there was broad involvement of MCP staff and the active engagement of DHCS quality consultants. Third, there was ongoing sharing among MCP peers of best practices and barriers.

This study has several limitations. The preintervention/postintervention design limits attribution of causality. In addition, the 9 MCPs that were studied volunteered to participate in the QIC and may have been motivated because of historical declines in hypertension control. Also, the intervention period was only 1 year, so long-term effects could not be assessed.

Nevertheless, hypertension control trends among both participating and nonparticipating MCPs were generally downward, suggesting that the QIC helped to improve outcomes at a system (ie, MCP) level. The results suggest that learning collaboratives can advance health care quality metrics with a moderate investment of resources. The shared learning may have contributed to the improved outcomes compared with the preceding period when plans were attempting improvement solely at an individual health-plan level.

DHCS has established mandatory learning collaboratives in the area of hypertension control, as well as in others. More research about such shared learning is needed to examine long-term effects, overcome potential cultural resistance, and develop sustainable business models.
